# Inositol Polyphosphate Kinases, Fungal Virulence and Drug Discovery

**DOI:** 10.3390/jof2030024

**Published:** 2016-09-06

**Authors:** Cecilia Li, Sophie Lev, Adolfo Saiardi, Desmarini Desmarini, Tania C. Sorrell, Julianne T. Djordjevic

**Affiliations:** 1 Centre for Infectious Diseases and Microbiology, The Westmead Institute for Medical Research, The University of Sydney, Westmead, NSW 2145, Australia; celi4752@uni.sydney.edu.au (C.L.); levsophie@gmail.com (S.L.); desmarini.desmarini@sydney.edu.au (D.D.); tania.sorrell@sydney.edu.au (T.C.S.); 2 Medical Research Council Laboratory for Molecular Cell Biology, University College London, London WC1E 6BT, UK; dmcbado@ucl.ac.uk; 3 Marie Bashir Institute for Infectious Diseases and Biosecurity, University of Sydney, Westmead, NSW 2145, Australia; 4 Westmead Hospital, Westmead, NSW 2145, Australia

**Keywords:** antifungal compounds, *Cryptococcus neoformans*, inositol polyphosphate kinases, inositol pyrophosphates, PP-IP_5_, fungal virulence, cryptococcal meningitis, drug discovery

## Abstract

Opportunistic fungi are a major cause of morbidity and mortality world-wide, particularly in immunocompromised individuals. Developing new treatments to combat invasive fungal disease is challenging given that fungal and mammalian host cells are eukaryotic, with similar organization and physiology. Even therapies targeting unique fungal cell features have limitations and drug resistance is emerging. New approaches to the development of antifungal drugs are therefore needed urgently. *Cryptococcus neoformans*, the commonest cause of fungal meningitis worldwide, is an accepted model for studying fungal pathogenicity and driving drug discovery. We recently characterized a phospholipase C (Plc1)-dependent pathway in *C. neoformans* comprising of sequentially-acting inositol polyphosphate kinases (IPK), which are involved in synthesizing inositol polyphosphates (IP). We also showed that the pathway is essential for fungal cellular function and pathogenicity. The IP products of the pathway are structurally diverse, each consisting of an inositol ring, with phosphate (P) and pyrophosphate (PP) groups covalently attached at different positions. This review focuses on (1) the characterization of the Plc1/IPK pathway in *C. neoformans*; (2) the identification of PP-IP_5_ (IP_7_) as the most crucial IP species for fungal fitness and virulence in a mouse model of fungal infection; and (3) why IPK enzymes represent suitable candidates for drug development.

## 1. Invasive Fungal Infections: The Clinical Burden

Invasive fungal infections (IFIs) are a major cause of global morbidity and mortality. The majority of IFIs worldwide are caused by *Aspergillus* spp., *Candida* spp., *Cryptococcus* spp., and *Pneumocystis jirovecii*. However, IFIs caused by emerging fungal pathogens, such as the yeast-like *Trichosporon* spp., *Rhodotorula* spp., and the moulds, *Scedosporium* spp., *Fusarium* spp., and *Zygomycetes* spp., are on the rise [1,2,3,4,5]. Due to a lack of international epidemiological and surveillance data, the effect of IFIs on human health is often under-reported [2,6] and consequently the true impact of IFIs on human health is not widely recognized.

Much of the burden of IFIs is on developing countries, which have high incidences of HIV/AIDS. According to a recent UNAIDS Global AIDS Report, 25.8 million people are estimated to be living with HIV in Sub-Saharan Africa, and the region accounts for almost 70% of new HIV infections world-wide [7]. In addition to HIV/AIDs patients, IFIs pose a significant threat to solid organ and hematopoietic stem cell or bone marrow transplant recipients. The US Centers for Disease Control and Prevention (CDC) has developed a list of the 20 most common opportunistic infections affecting immunocompromised individuals. Of these, 5 are caused by fungi [8]. IFIs are also an enormous economic liability to the global health system: a US study found that transplant patients with an IFI stayed in hospital an additional 19 days longer than patients without an IFI, resulting in an excess of ~USD 55,000 for hospital costs/patient [9]. Furthermore, there is a 5-fold increase in the mortality rate of these patients [9].

## 2. Molecular Targets of Antifungal Drugs and Drug Limitations

As fungi are eukaryotes, the number of selective drug targets is more limited than in the case of prokaryotes. Most marketed drugs (e.g., amphotericin B (AMB), azoles, terbinafine) target the unique fungal plasma membrane sterol, ergosterol, or its biosynthetic pathway, while the echinocandins and 5-flucytosine (5-FC) inhibit the unique cell wall and DNA synthesis, respectively. However, current antifungal therapies have limitations, which compound the adverse public health impact of IFIs. These limitations include an incomplete antifungal spectrum, toxicity, poor bioavailability, poor solubility or stability, and/or high cost. Although well tolerated, the echinochandins are ineffective in treating IFIs caused by *Cryptococcus neoformans* [10,11,12,13]. Multi-azole resistant *Aspergillus fumigatus* has been reported [14,15,16,17] and azole and echinocandin resistance has been observed in non-albicans *Candida* spp. [18,19,20]. Despite this, fluconazole resistance in *Candida* spp. was the only mention of antifungal drug resistance in the 2014 WHO global surveillance report [21].

For the treatment of meningitis caused by *C. neoformans*, guidelines issued by the World Health Organization and the Infectious Diseases Society of America, recommend induction therapy with AMB and 5-FC for 2 weeks, followed by maintenance therapy with the azole drug, fluconazole, for 8 weeks [22,23]. In resource limited countries where 5-FC may be frequently unavailable, it is recommended that adults are started on a high-dose fluconazole regime with AMB if the latter is available [22]. However, Day et al. published that AMB and fluconazole combination therapy did not increase patient survival in a Vietnamese cohort and called for greater access to 5-FC; a call echoed by others [24,25,26,27]. Infection relapse can also occur, particularly in patients who are not receiving maintenance therapy or antiretroviral therapy [28]. It is also of great concern that fluconazole-resistant *C. neoformans* has been reported in Sub-Saharan Africa and South East Asia, regions where the disease burden is the highest [29,30,31,32,33]. It is imperative to develop new drugs to combat these infections. This can be achieved either by repurposing old drugs which are useful in the treatment of other infectious microbes or by developing new classes of antifungal agents. To facilitate the development of new drugs, an understanding of the mechanisms that fungi employ to establish infection and cause disease in the human host is of paramount importance.

## 3. *Cryptococcus neoformans*: A Study Model for Virulence and Drug Development

*C. neoformans* is an opportunistic basidiomycetous yeast pathogen causing serious morbidity and mortality in immunocompromised individuals. Infection is acquired by inhalation of infectious propagules (spores or small desiccated yeast cells) into the lungs and manifests as pneumonia-like symptoms. Yeast cells can further disseminate via the blood and cross the blood brain barrier, resulting in life-threatening meningoencephalitis. Up to one million new cases of cryptococcal meningitis in people living with HIV/AIDs and around 600,000 deaths have been reported to occur annually [34]. Even with access to antiretroviral and antifungal therapy, mortality rates due to cryptococcal meningitis remain at 20%–40% [35,36,37,38]. *C. neoformans* is also a powerful model for elucidating the mechanisms that fungi use to cause disease: it is a budding yeast that is easy to grow, with minimal nutritional requirements and a defined mating cycle [39,40,41,42]; it produces a suite of virulence traits that are easily measured; and its haploid genome is highly amenable to targeted gene deletion, which can be combined with mating crosses to increase the number of gene deletions within the same genome [43,44]. Furthermore, a number of vertebrate and invertebrate infection models have been developed to address the impact of gene deletion on pathogenesis and virulence and to assess drug efficacy [45,46,47,48,49], also reviewed in [50,51].

*C. neoformans* virulence is multifactorial and includes the production of capsule, melanin, urease, Sec14-dependent phospholipase B1 (Plb1), the formation of titan cells and high temperature tolerance [52,53,54,55,56,57,58,59,60,61,62]. While some of the *C. neoformans* virulence determinants are unique, others contribute to virulence in other fungal pathogens, e.g., phospholipase B in *Candida albicans* [63,64], urease in *Coccidioides posadasii* [65], melanin and the extracellular polysaccharide (galactosaminogalactan) in *Aspergillus fumigatus* [66,67]. Production of virulence factors in *C. neoformans* is regulated by key signaling pathways, including the calcineurin, protein kinase C/mitogen-activated protein kinase (Pkc1/Mpk1), cyclic adenosine monophosphate/protein kinase A (cAMP/Pka1), high osmolarity glycerol (HOG), and Rim101 pathways [68,69,70,71,72,73,74]. Phospholipase C (Plc1)-dependent signalling also contributes to the fitness and virulence of *C. neoformans* [75], and the next section describes how this pathway was elucidated and characterized in this pathogen.

## 4. Signaling via Plc1 in *C. neoformans*: The Role of Inositol 1,4,5-Trisphosphate (IP_3_)

We established that phosphatidylinositol (PI)-specific phospholipase C1 (Plc1)—the homologue of Plc1 in *Saccharomyces cerevisae* [76,77,78] and mammalian phospholipase C (PLC-δ) [79] is essential for the virulence of *C. neoformans* [75]. In a mouse inhalation model, we found that infection with the cryptococcal *PLC1* deletion mutant (*plc1*Δ) was cleared from the lung 14 days post-infection, and that the mutant failed to disseminate to the brain [75]. Furthermore, *PLC1* deletion resulted in the attenuation of several key virulence factors: growth at 37 °C, production of melanin (and capsule under some growth conditions) and secretion of phospholipase B1 (Plb1). Given that Plb1 accumulated in the membrane in *plc1*Δ, we hypothesized that Plc1 also plays a role in releasing membrane-associated Plb1 from its glycosylphosphatidylinositol (GPI) anchor. However, this was not demonstrated directly. *plc1*Δ also had a cell wall integrity defect and was hyper-susceptible to all antifungal agents used in the treatment of cryptococcosis [75].

To investigate how Plc1 regulates cellular function, we established that recombinant cryptococcal Plc1 preferentially hydrolyses PI-4,5-bisphosphate (PIP_2_) to produce two signaling molecules, namely, inositol 1,4,5-trisphosphate (IP_3_) and diacylglycerol (DAG) [80]. In agreement with this finding, the *plc1*Δ mutant accumulated PIP_2_ and had reduced IP_3_ levels, as demonstrated using a radiometric IP_3_ assay [80]. In addition to Plc1, DAG is produced by the enzyme inositol-phosphorylceramide synthase-1 (Ipc1) and functions as an activator of cryptococcal Pkc1 [81]. Pkc1 acts upstream of the MAP kinase cell wall integrity pathway (Bck1/Mkk2/Mpk1), but also regulates melanization and cell wall homeostasis independently of the MAPK cascade [81,82]. Given the degree of phenotypic similarity between *plc1*Δ and *pkc1*Δ, including compromised melanin production and a cell wall integrity defect, and that cell wall perturbing agents fail to activate the Pkc1/Mpk1 pathway in *plc1*Δ, we deduced that Plc1-derived DAG contributes to Pkc1 activation [75].

In mammalian cells, IP_3_ derived from PLC-δ interacts with IP_3_ receptors in the endoplasmic reticulum, causing a release of intracellular calcium and an activation of calcineurin [83,84,85,86]. Fungal homologs of the mammalian IP_3_ receptor have not been identified, although IP_3_-mediated calcium release has been demonstrated in hyphal tips of filamentous fungus *Neurospora crassa* [87]. We observed that *plc1*Δ and the cryptococcal calcineurin deletion mutant (*cna1*Δ) shared significant phenotypic similarity under a range of stress conditions, suggesting that Plc1-derived IP_3_ could activate calcineurin in *C. neoformans*. However, we demonstrated that the simultaneous inactivation of Plc1 and calcineurin was more detrimental to cryptococcal growth than the inactivation of either calcineurin or Plc1 alone, suggesting that cryptococcal Plc1 activates effectors other than calcineurin. We concluded that Plc1 and the calcineurin pathway function in parallel to regulate a common set of phenotypes, including the ability to grow at 37 °C, cell wall integrity and cell morphology [68,80].

## 5. Delineating the IP Biosynthesis Pathway in *C. neoformans*

The cellular fate of Plc1-derived IP_3_ has been determined in non-pathogenic yeast and mammalian cells. The genomes of these organisms encode kinases, which use ATP to phosphorylate IP_3_ and other inositol polyphosphates (IP), to generate a structurally diverse family of IP species. Thus, in addition to its role as a secondary messenger, IP_3_ serves as a precursor for the synthesis of more complex IP. IP species are comprised of a six-carbon *myo*-inositol ring to which phosphates are covalently attached at various positions by inositol polyphosphate kinases (IPK). There are essentially two types of IP: those with single covalently attached phosphates and those with a mixture of single and diphosphate groups (inositol pyrophosphates, PP-IP). PP-IP contain “high-energy” phosphate bond(s), and are thought to undergo a rapid turnover in eukaryotic cells [88]. The IP biosynthesis pathway has been extensively studied in a model ascomycetous yeast, *S. cerevisiae* (reviewed in [89,90,91]) and our recent characterization of the pathway in *C. neoformans* (described forthwith) is the first in a fungal pathogen [80,92,93].

**Figure 1:**
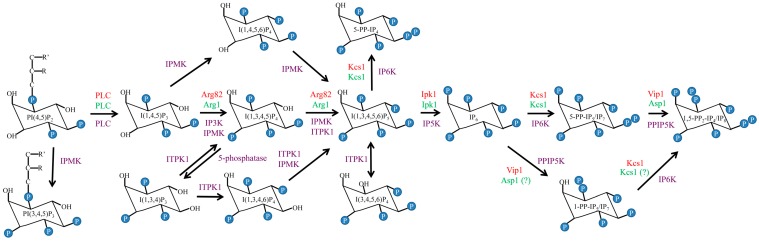
Comparison of the phospholipase C1/inositol polyphosphate kinase Plc1/IPK pathways in yeast and humans. Enzymes in *C. neoformans*, *S. cerevisiae* and humans are marked in green, red and purple, respectively.

Using a homology search, we identified a number of putative IPK-encoding genes in the *C. neoformans* var *grubii* (wild-type strain H99 [WT], serotype A) genome. These included Arg1 and Arg2 [80], which are most similar to *S. cerevisiae* Arg82, a dual-specificity kinase phosphorylating Plc1-generated I(1,4,5)P_3_ to I(1,3,4,5)P_4_, and I(1,3,4,5)P_4_ to I(1,3,4,5,6)P_5_ [94,95], and the putative I(1,3,4,5,6)P_5_ kinase, Ipk1 [93] (Figure 1). We also identified two putative inositol pyrophosphate synthases, Kcs1 and Vip1 [92], which were predicted to add phosphates to the existing phosphates at positions 5 and 1, respectively, of the inositol ring. Similar to the fission yeast, *Schizosaccharomyces pombe* [96], Vip1 is referred to as Asp1 in the cryptococcal database.

**Figure 2:**
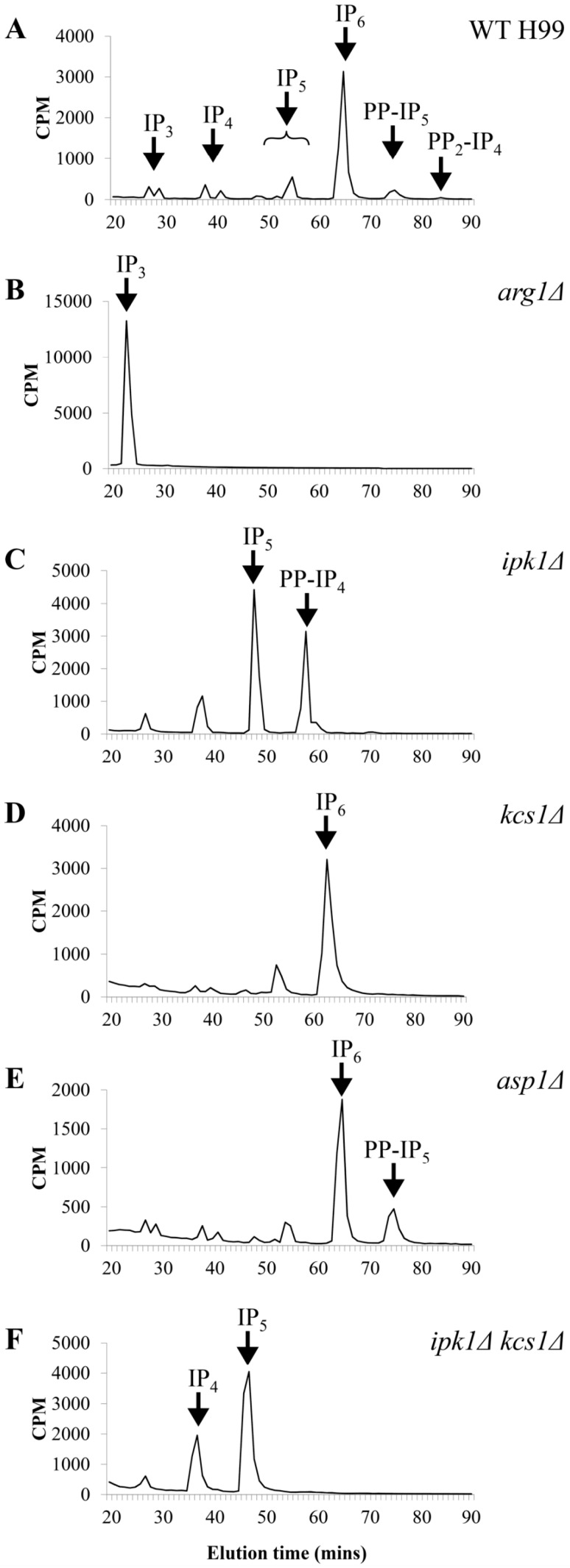
Comparison of the IP profiles of (**A**) WT [92,93]; (**B**) *arg1*Δ [92]; (**C**) *ipk1*Δ [93]; (**D**) *kcs1*Δ [92]; (**E**) *asp1*Δ [92] and (**F**) *ipk1*Δ *kcs1*Δ [93]. Lysates prepared from [^3^H]-myo-inositol-labeled cells were subjected to anion-exchange HPLC analysis.

To confirm the function and substrate specificity of the IPK enzymes identified in *C. neoformans*, we determined the IP profiles of the corresponding deletion mutants using strong anion exchange (SAX)-HPLC (summarized in Figure 2 from publications [92,93]). Firstly, we confirmed that Arg1 is the major IP_3_ kinase in *C. neoformans*, since the *ARG1* deletion mutant (*arg1*Δ) accumulated IP_3_ and was deficient in more complex IP species [80,92] (Figure 2B, Table 1). Despite the presence of the conserved IPK amino acid sequence motif, P-x-x-x-D-x-K-x-G [94], the *arg2*Δ mutant did not accumulate IP_3_ and was phenotypically identical to WT, demonstrating that Arg1 and Arg2 are not functionally redundant [80]. Although *ARG2* most likely arose from *ARG1* by gene duplication, its role in cellular function is not apparent [80].

HPLC analysis of the IP profiles of the cryptococcal *ipk1*Δ and *kcs1*Δ mutants established that Ipk1 and Kcs1 are IP_5_ and IP_6_ kinases, respectively (Figure 2C,D) [92,93]. However, in the absence of Ipk1, Kcs1 also functions as an IP_5_ kinase: IP_5_ accumulates in the *ipk1*Δ mutant and a new pyrophosphate species, 5-PP-IP_4_, is created by Kcs1 via the addition of phosphate to the phosphate at position 5 (Figure 2C,F) [93]. When functional Ipk1 was present, Kcs1 phosphorylated IP_6_ to create 5-PP-IP_5_ (IP_7_). In the absence of Kcs1, PP-IP_5_ and PP_2_-IP_4_ (IP_8_) were not detected, suggesting that Kcs1 is the main IP_6_ kinase/PP-IP_5_ synthase in *C. neoformans* (Figure 2D, Table 1) [92].

Unlike other IPKs, mammalian and fungal Vip1/Asp1 enzymes have a dual domain structure comprising of an N-terminal kinase and a C-terminal histidine acid phosphatase-like domain. Except for the aspartate residue adjacent to the second histidine in the consensus active site, the key histidine and arginine residues are present in the Vip1 acid phosphatase-like domain [97]. Several studies demonstrate that the C-terminal domain of Vip1 has a negative effect on inositol pyrophosphate output by the N-terminal kinase domain, presumably via direct phosphate hydrolysis [96,97,98,99]. Interestingly, the acid phosphatase-like domain of Vip1 has also been implicated in binding the phospholipid, phosphatidylinositol (3,4,5)-trisphosphate [100]. We established that, similar to other Vip1 enzymes, cryptococcal Asp1 phosphorylates the Kcs1-derived product, 5-PP-IP_5_, to produce 1,5-PP_2_-IP_4_ (Figure 1). This is indicated by the absence of PP_2_-IP_4_ in the *asp1*Δ mutant (Figure 2E, Table 1) [92].

In addition to phosphorylating 5-PP-IP_5_, Vip1 enzymes also phosphorylate IP_6_ in vivo and in vitro [97,101]. However, the 1-PP-IP_5_ product of Vip1 is not easily detectable in vivo, presumably due to its rapid hydrolysis by phosphatases, including the C-terminal domain of the Vip1 protein itself [88,102]. In *S. cerevisiae*, Vip1-derived PP-IP_5_ was detectable by HPLC, but only when the polyphosphate phosphatase, Ddp1, and Kcs1 were absent (*kcs1*Δ *ddp1*Δ mutant) [101]. Interestingly, the cryptococcal genome does not encode a Ddp1 homolog. However, we did identify a close homolog of *S. cerevisiae* Siw14, which specifically dephosphorylates Kcs1-derived 5-PP-IP_5_, to modulate PP-IP metabolism [103].

## 6. The Contribution of IP/PP-IP Species to Cryptococcal Cellular Function

To determine the contribution of individual IP species to cryptococcal cellular function and virulence, we compared the phenotypic profiles of the entire IPK deletion mutant set [75,80,92,93] (summarized in Table 2). Overall, the mutants could be ranked as follows: *plc1*Δ *> arg1*Δ *> kcs1*Δ *> ipk1*Δ *> asp1*Δ, with *plc1*Δ having the most severe phenotypic defect. Similar to *plc1*Δ, growth of *arg1*Δ was compromised at 37 °C [80]. Both mutants exhibited a cell separation defect, enlarged vacuoles (more pronounced in *arg1*Δ), small capsules and a thickened cell wall. In contrast to *arg1*Δ, *plc1*Δ had diminished endocytic activity [80]. The virulence of *plc1*Δ and *arg1*Δ was attenuated in *Galleria mellonella* larvae incubated at the permissive mutant growth temperature of 30 °C. However, larvae infected with the *plc1*Δ mutant survived longer than those infected with *arg1*Δ [80].

Cell wall integrity and production of urease, melanin and mating filaments was compromised in the *plc1*Δ, *arg1*Δ and *kcs1*Δ mutants [75,80,92]. Defects in cell wall integrity and urease production were also observed in *ipk1*Δ, but to a lesser extent than in *arg1*Δ or *kcs1*Δ [93]. Melanization in *plc1*Δ, *arg1*Δ and *kcs1*Δ was assessed by examining colony color in the presence of the laccase substrate, l-Dopa, over a 3–4 day period. In a more quantitative approach, we assessed laccase activity in *ipk1*Δ and *ipk1*Δ *kcs1*Δ cells following a shift to laccase-inducing conditions (3–6 h in a minimal medium without glucose). Surprisingly, we discovered that laccase activity in these mutants was significantly reduced compared to WT [93], despite WT-like melanization in *ipk1* Δ and *ipk1* Δ *kcs1* Δ. This suggests that the presence of low laccase activity over several days is sufficient to produce WT-like melanization in the mutants.

The virulence of *kcs1*Δ and *ipk1*Δ was also compromised: 100% and 80% of mice infected with *kcs1*Δ and *ipk1*Δ, respectively, survived the infection over a 50-day time course [93]. In contrast, *asp1*Δ was phenotypically identical to WT, and just as virulent as WT in mice, suggesting that PP_2_-IP_4_ plays a minor role in cryptococcal homeostasis and virulence [92]. In other fungal species, including *Schizosaccharomyces pombe*, *Aspergillus nidulans* and *Ustilago maydis*, Vip1/Asp1 plays a role in polarized growth and the dynamics of the microtubular cytoskeleton [98]. Possible reasons for the varying importance of PP_2_-IP_4_ among the different fungal species remain to be elucidated.

Given that *asp1*Δ (1,5-PP_2_-IP_4_-deficient) is as virulent as WT *C. neoformans* [92], the common phenotypic defects of the *plc1*Δ, *arg1*Δ, *ipk1*Δ and *kcs1*Δ mutants are likely to be caused by the absence of 5-PP-IP_5_. We therefore postulated that 5-PP-IP_5_ is the crucial metabolite responsible for numerous cellular functions in *C. neoformans* [92]. Interestingly, the *ipk1*Δ mutant was the least affected by the absence of 5-PP-IP_5_, exhibiting a more robust phenotype than other 5-PP-IP_5_-deficient strains [93]. We rationalized that the accumulation of Kcs1-derived 5-PP-IP_4_ in *ipk1*Δ contributed to the fitness of this mutant by fulfilling some of the functions of its pyrophosphorylated cousin, 5-PP-IP_5_ [93].

## 7. 5-PP-IP_5_ Plays a Critical Role in Cryptococcal Virulence

A hallmark phenotype of the *kcs1*Δ mutant is its mucoid appearance and large capsules after being cultured on Sabouraud agar [92]. However, this hyper-encapsulation phenotype was not apparent during the infection of the mouse lung [92]. Similar to *plc1*Δ, *kcs1*Δ was avirulent in a mouse inhalation model [92]. However, the *kcs1*Δ infection differed from that of *plc1*Δ. As opposed to *plc1*Δ, which was cleared from the mouse lung 14 days post-infection [92], *kcs1*Δ established a residual lung infection over a 50 day period that failed to disseminate to the central nervous system [92]. The reduced proliferation of *kcs1*Δ in the lung tissue may be attributed to its inability to utilize non-glucose carbon sources [92]. Given that the lung is a low glucose environment, this phenotype is likely to have a negative impact on cellular fitness and subsequently on virulence. These metabolic differences suggest that WT, but not *kcs1*Δ, actively expresses enzymes involved in the utilization of carbon sources other than glucose, (e.g., lactate and fatty acids), which require mitochondrial function. To investigate this further, we performed a high throughput gene expression analysis and showed that our data was supportive of metabolic dysfunction in the absence of PP-IP_5_. Genes encoding proteins involved in glycolysis were more highly expressed in *kcs1*Δ than in WT, while the expression of genes encoding enzymes involved in the utilization of alternative carbon sources (citric acid and glyoxylate cycles, gluconeogenesis, and fatty acid β-oxidation) was reduced in *kcs1*Δ, suggesting that mitochondrial function is suppressed in *kcs1*Δ [92].

To further understand the pathology of *kcs1*Δ infection, we compared the immune response in the mouse lung during infection using cytokine profiling [92]. The results showed that *kcs1*Δ elicited a much weaker cytokine response: lower interferon-gamma (IFN-γ), tumor necrosis factor (TNF), monocyte chemoattractant protein-1 (MCP-1), IL-6 and IL-4, and delayed production of IL-2 and IL-17A, compared to WT [92]. It is known that mannoproteins present in the cryptococcal polysaccharide capsule and cell wall are highly immunogenic [104,105,106,107]. We observed reduced recognition of *kcs1*Δ by monocytes in vitro, which correlated with diminished mannoprotein exposure at the *kcs1*Δ surface, as determined by concanavalin A binding [92]. Furthermore, transcription of mannoprotein-encoding genes was reduced in *kcs1*Δ, as indicated by high throughput gene expression analysis [92]. Taken together, our data suggested that the persistence of a low-grade, asymptomatic *kcs1*Δ mutant infection is due, in part, to reduced monocyte recruitment to the sites of *kcs1*Δ infection, and the reduced uptake of *kcs1*Δ by both lung resident macrophages and monocytes infiltrating from the vasculature [92].

## 8. The Plc1/IPK Pathway: A Signaling or a Metabolic Pathway?

The Plc1/IPK pathway has the features of both a metabolic and a signaling pathway. On the one hand the Plc1/IPK pathway is an anabolic pathway where sequentially acting kinases use the energy of ATP to synthesize IP and PP-IP of increasing complexity. However, some of the IP products of the pathway have roles in signaling.

### 8.1. Features of a Metabolic Pathway

In *C. neoformans* and *S. cerevisiae*, the Plc1/IPK pathway is constitutively active, maintaining stable levels of each IP species, with IP_6_ being the most abundant (Figure 2A, Table 1). Furthermore, the role of this pathway in *C. neoformans* is not limited to the regulation of virulence determinants in response to host-derived stimuli, but includes fungal fitness, as evidenced by the delayed growth of *plc1*Δ and *kcs1*Δ under optimal conditions, as well as under conditions of stress [75,92]. In yeast and mammalian cells, biosynthesis of PP-IP_5_ by Kcs1 is tightly linked to ATP availability [94,108]. Due to the rapid hydrolysis of PP-IP_5_ by intracellular phosphatases [88], its intracellular concentration is not maintained at the same level following ATP depletion, but drops rapidly. During phosphate deprivation in *S. cerevisiae*, PP-IP_5_ levels are decreased in correlation with the reduction in ATP [109,110]. Thus, PP-IP_5_ appears to serve as an indicator of intracellular energy status, rather than as a classical secondary messenger.

### 8.2. Features of a Signaling Pathway

A signaling pathway is defined as one which is activated by an extracellular stimulus. The signal is then propagated intracellularly via secondary messengers, which fluctuate in response to a stimulus. The secondary messengers then engage signaling components downstream to elicit physiologically relevant responses. IP/PP-IP can act as secondary messengers. Stimulus-induced changes in IP/PP-IP levels in mammalian and fungal cells have been demonstrated in several instances. Perhaps the best studied example is Plc1-mediated IP_3_ release in mammalian cells following ligand stimulation, leading to an influx of calcium from the ER and the activation of calcineurin [83,84,85,86]. In the filamentous fungus, *Neurospora crassa* membrane stretch at the hyphal tip due to growth is thought to activate PLC, triggering IP_3_ production. IP_3_ induces calcium release from vesicles localized at the hyphal tip allowing the establishment of a calcium gradient, which is essential for tip expansion [87]. IP_3_ also triggers calcium release from vacuoles in *S. cerevisiae* [111], *N. crassa* [87,112] and *C. albicans* [113].

In mammalian cells I(3,4,5,6)P_4_ inhibits Ca^2+^-activated chloride channels in the plasma membrane. These ion channels are required for salt and fluid secretion from epithelial cells, insulin secretion from pancreatic β-cells, smooth muscle contraction and neurotransmission [114,115,116,117]. The intracellular concentration of I(3,4,5,6)P_4_ increases during sustained stimulation by phospholipase C agonists [118]. In mammalian cells, I(1,3,4)P_3_ derived from PLC via I(1,4,5)P_3_ stimulates the production of I(3,4,5,6)P_4_ by inositol 1,3,4-triphosphate 5/6 kinase ITPK1 (Figure 1) [119,120]. Chamberlain et al. hypothesized that the function of primordial ITPK1 was to promote the biosynthesis of highly phosphorylated IP species. However, in mammalian cells this function has evolved to accommodate a signaling role for I(3,4,5,6)P_4_ [120].

PP-IP levels also fluctuate in response to stress: in mammalian cells, hyperosmotic (0.2 M sorbitol), heat (42 °C) and cold (33 °C) stresses triggered an increased production of PP_2_-IP_4_ [121,122]. In contrast, neither hyperosmotic nor heat stress caused significant fluctuations in IP/PP-IP levels in *S. cerevisiae* [121]. However, in *S. pombe* the IP_6_ level tripled within minutes of exposure to high salt concentration (0.7 M KCl) [123]. In agreement with the role of IP/PP-IP in stress tolerance, Worley et al. demonstrated that activation of the stress response (by heat and high salt) in *S. cerevisiae* requires Kcs1 and Vip1-derived PP-IP. These molecules act in parallel with the TORC1 pathway to control the activity of the class I histone deacetylase Rpd3L [124].

Taken together, the data above demonstrates that specific environmental stimuli have an impact on intracellular IP/PP-IP concentration in several experimental models. However, the relative stability of the IP profile in fungal and mammalian cells suggests that the biosynthesis of highly phosphorylated IP is a constitutive process, functioning to maintain cellular function even in the absence of specific extracellular stimuli. In fungal pathogens, including *C. neoformans*, the dynamic changes in individual IP/PP-IP species that occur in response to various stress conditions require investigation. However, such investigation is hindered by the technical difficulty encountered in separating and quantifying these molecules, and also by the lack of commercially available IP standards.

## 9. How IP/PP-IP Regulate Cellular Function

IP species, and PP-IP_5_ in particular, regulate numerous and diverse cellular processes including endocytosis, vesicular trafficking, vacuole formation, autophagy, maintenance of telomere length, apoptosis, cytoskeletal organization, DNA repair and neutrophil-dependent antimicrobial defense (reviewed in [71,89,90,125]). So how do IP achieve this? Do they have specific individual effects on each function or do they function upstream of a pathway, which itself regulates numerous functions? Evidence obtained from yeast and mammalian systems supports both hypotheses, with IP/PP-IP serving as effector molecules to exert allosteric protein regulation (IP and PP-IP), or as phosphate donors to pyrophosphorylate proteins (PP-IP).

In a more recent discovery, PP-IP were shown to allosterically regulate yeast, plant and mammalian proteins containing an SPX domain [126]. SPX domains (135–380 residues) are found in proteins involved in the regulation of phosphate homeostasis, which, in *S. cerevisiae*, include low-affinity phosphate transporters, components of the vacuolar polyphosphate polymerase complex and the cyclin-dependent kinase inhibitor, Pho81 (reviewed in [127]). In *S. cerevisiae*, Kcs1-derived PP-IP_5_ directly stimulates the synthesis of inorganic polyphosphate chains in vacuoles by binding to the SPX-domain of the polyphosphate polymerase complex [126]. The SPX domain of the cyclin-dependent kinase inhibitor, Pho81, is likely to mediate inhibition of CDK Pho85 under phosphate starvation conditions, leading to the activation of the PHO pathway [128]. I(1,4,5,6)P_4_ is also an allosteric regulator of histone deacetylase class I complexes in mammalian cells [129,130].

As an alternative to allosteric binding, PP-IP were shown to pyrophosphorylate proteins by virtue of their “high energy” pyrophosphate group: the free energy of hydrolysis of the pyrophosphate bond in PP-IP_5_ is 6.6 Kcal/mol, which is higher than that of ADP [131], while for PP_2_-IP_4_ it is estimated to be higher than that of ATP (7.3 Kcal/mol), due to the alleviation of electrostatic and steric constraints [132,133]. Phosphate transfer by PP-IP_5_ is non-enzymatic, but magnesium-dependent, and requires the target protein to be pre-phosphorylated on serine. Proteins encoding stretches of serines flanked by acidic amino acids or regions containing multiple lysines are good targets for PP-IP_5_-mediated pyrophosphorylation [133]. It has been shown that casein kinase 2 (CK2) efficiently primes proteins for pyrophosphorylation by PP-IP_5_ in vitro [134]. In *S. cerevisiae*, PP-IP_5_ was shown to suppress the expression of glycolytic genes via pyrophosphorylation of the transcription factor Gcr1 [135]. Similar to other post-translational modifications, PP-IP-mediated pyrophosphorylation represents a unique mode of intracellular communication. It should be kept in mind though that the generation of pyrophosphorylated proteins by PP-IP_5_ is yet to be observed in vivo. Further exploration of this phenomenon will allow us to distinguish which of the pleiotropic effects of PP-IP_5_ are conveyed via protein pyrophosphorylation versus allosteric binding.

## 10. The Plc1/IPK Pathway as a Target for Antifungal Drug Development

Given the significant impact that the loss of PP-IP_5_ has on cryptococcal virulence in animal models, the Plc1/IPK pathway represents an attractive avenue for antifungal drug development. From a clinical perspective, targeting enzymes upstream of PP-IP_5_, rather than the PP-IP_5_ itself, is the only suitable option. While IP and PP-IP have critical roles in regulating diverse cellular processes in the pathogen itself, the same metabolites are produced in the host and play similar essential roles. Targeting fungal-derived IP/PP-IP could therefore have detrimental side effects. Although IP/PP-IP are ubiquitous secondary metabolites in all eukaryotes, the primordial Plc1/IPK pathway, responsible for their production in fungi, has evolved into a more complex one in mammalian cells over millions of years. The absence of redundancy in the fungal pathway, the low homology shared by fungal and mammalian IPK enzymes and the discovery of naturally-occurring inhibitors of these kinases further support their suitability for future drug development. In addition, the cryptococcal mutants, *plc1*Δ, *arg1*Δ, *ipk1*Δ and *kcs1*Δ, are all hypersusceptible to antifungal agents, suggesting that inhibitors against each of the respective fungal enzymes could act synergistically with marketed antifungal drugs against wild-type pathogens to achieve a more favorable treatment outcome.

Compared to the cryptococcal IP biosynthesis pathway, the mammalian pathway is more complex and includes multiple enzymes with similar catalytic activity. For example, while Arg1 in *C. neoformans* is the only enzyme able to convert IP_3_ to IP_4_, three IP3K isoforms (IP3K-A, IP3K-B and IP3K-C) and the inositol polyphosphate multikinase (IPMK) catalyze this reaction in humans (reviewed in [89,91]) (Figure 1). Similarly, mammals express three isoforms of IP6K, with each having a specialized physiological function (reviewed in [90]).

The human repertoire of IPKs also includes an enzyme, which is structurally and functionally unique to mammalian cells: hITPK1 (inositol 1,3,4-triphosphate 5/6 kinase) mediates the transfer of phosphate between different IP molecules (“intersubstrate” transfer). This mechanism is instrumental in the PLC-dependent increase in I(3,4,5,6)P_4_ levels: PLC-generated I(1,4,5)P_3_ is converted to I(1,3,4)P_3_, which stimulates I(1,3,4,5,6)P_5_ dephosphorylation by ITPK1 to produce biologically active I(3,4,5,6)P_4_ (Figure 1) [119,120,136]. Although ITPK1 homologs are present in soybean and *Entamoeba histolytica*, this mechanism is only functional in mammalian cells. A study by Chamberlain et al. pinpoints structural differences between human and *E. histolytica* enzymes, potentially responsible for the unique features of hITPK1 [120,137]. Human ITPK1 has no homologs in *C. neoformans*, *C. albicans* or *A. fumigatus*.

In contrast to mammalian cells, the cryptococcal IP biosynthesis pathway is non-redundant and all three sequentially acting enzymes, Arg1, Ipk1 and Kcs1, are required for the production of PP-IP_5_. The presence of IPK enzymes in other medically important pathogens, including *Candida* spp. and *Aspergillus* spp., supports their exploitation as panfungal drug targets.

## 11. Structural Studies of IPKs

The three dimensional structures of several IPK enzymes, including Arg82 from *S. cerevisiae* (Ipk2) [138], the hybrid IP6K/IP3K from *E. histolytica* [139], IPMKα from *Arabidopsis thaliana* [140], rat and human IP3K [141,142], and ITPK from *E. histolytica* and human [120,137] have been solved. Structural comparison and substrate modelling were also used to establish the basis for IPK substrate specificity. IP3K, IPMK and IP6K enzymes share a conserved amino acid motif P-x-x-x-D-x-K-x-G in their inositol binding region (Pfam family PF03770). They are predicted to assume the same overall fold despite their low sequence conservation [138]. The sequence variation allows these enzymes to discriminate between different IP species through a combination of steric exclusion and the requirement for the occupation of specific phosphate binding pockets. Some IPK enzymes contain additional domains or insertions, which have a physiological role. For example, a poly-D loop insertion in yeast Ipk2 was shown to facilitate Ipk2 interaction with the transcription factors, ArgRI and Mcm1 [143]. Thus, yeast Ipk2 (alias ArgRIII/Arg82) performs a unique second function, distinct from IP generation, which is to act as a stabilizer of a transcriptional regulatory complex involved in arginine metabolism [144]. Arg1 in *C. neoformans* contains a similar polyD/E loop insertion. However, this region is not conserved in the mammalian IPMK enzymes, suggesting that its function is restricted to yeast IPK enzymes.

Additional evidence of drug-exploitable differences in IPK active sites comes from a study performed by Wang et al. [145]. This group found that, in addition to the IP catalytic pocket, the surface of the kinase domain of mammalian PPIP5K (Vip1) contains a second unique substrate binding site with greater flexibility for substrate accommodation. This second site functions to deliver substrate to the catalytic site in a two-step binding process, which is the first of its kind to be described in small molecule kinases.

Differences in the structure of the inositol binding domain in human IP3K and *S. cerevisiae* Ipk2 were also identified. While the overall conformation is comparable in both enzymes, the inositol-binding domain is smaller in Ipk2 and contains only two of the five α-helices present in IP3K, suggestive of substrate accommodation restrictions in Ipk2 [138]. Taken together, these differential properties of IPK enzymes will potentially allow the development of inhibitors customized specifically for the fungal enzymes.

## 12. Advances in Identifying IPK Inhibitor Specificity

Pharmacological approaches taken towards understanding the mechanisms of action of IPK enzymes have led to the discovery of several IPK inhibitors that could serve as lead compounds for future drug development. Purine-based IP3K inhibitors were identified as a means to understand how mammalian IP3K regulates intracellular calcium metabolism [146]. Purine-based inhibitors were favored over inositol polyphosphate-based inhibitor analogues because the latter are highly charged and, therefore, unable to be delivered into cells without a carrier. In contrast, the purine-based inhibitors reported by Chang et al. are less negatively charged and thus can more easily penetrate cellular membranes, a property which is essential if these inhibitors are to be used for clinical purposes.

Padmanabhan et al. demonstrated that a purine analogue N^2^-(m-(trifluoromethyl)benzyl) N^6^-(p-nitrobenzyl) purine (TNP), selectively inhibits both human IP3K and IP6K enzymes by competing with ATP binding [147]. In vitro, the selectivity of TNP for IP6K was significantly higher than for IP3K (IC_50_ 18 vs. 0.47 µM). This difference is expected to be even more pronounced in vivo due to the relatively low affinity of IP6K for binding ATP. TNP has been used successfully to mimic IP6K (*KCS1*) deletion in *S. cerevisiae*: the addition of TNP to wild-type cells caused fragmentation of vacuoles, a typical feature of the *kcs1*Δ mutant [147,148].

Natural and synthetic plant polyphenolics have also been shown to inhibit all three IP3K isoforms, as well as IPMK in mammalian cells [149], but to varying degrees: the synthetic polyphenol, aurintricarboxylic acid (ATA), was the best inhibitor of all enzymes, while naturally occurring chlorogenic acid (a bioactive ingredient found in coffee) was specific to IPMK [149]. All inhibitors displayed non-competitive inhibition with respect to IP_3_-binding and a mixed-type inhibition with respect to ATP-binding. Mutagenesis studies showed that both the calmodulin binding and the IP_3_-binding domains in IP3K interact with the inhibitor. The absence of these domains in IPMK and the presence of a unique insertion in IPMK were found to be important for the selectivity differences observed for IP3K inhibition.

ATA and chlorogenic acid have been reported to have antiproliferative activity [150,151,152]. In addition, derivatives of chlorogenic acid have been described as having antifungal activity against *C. neoformans* and *C. albicans* [153]. Examples of other polyphenolic compounds which inhibit IPKs [149] and also have antifungal activity are provided in Table 3. These promising results suggest that polyphenolics, such as chlorogenic acid, have multipurpose functions and could be used in synergy with drugs to treat cancer and/or fungal infections. In summary, these studies provide proof-of-concept that selective inhibitors of fungal IPKs homologues either occur in nature or can be developed.

## 13. Conclusions

In summary we have characterized the Plc1/IPK pathway for the first time in a fungal pathogen of great significance to human health, and have demonstrated the important contribution of the pyrophosphate product, PP-IP_5_, to fungal fitness and pathogenicity in animal infection models. As the technology required to detect and synthesize IP evolves, it will soon be possible to fully elucidate the mechanism of action of PP-IP_5_ in fungi. The Plc1/IPK pathway in fungi has evolved into a more complex pathway in multi-cellular organisms, providing opportunities for drug development. These drugs could act alone to inhibit IPK enzymes, or synergistically with currently marketed antifungals to reduce side effects and provide more favorable treatment outcomes. By ignoring minimalist primordial pathways such as the Plc1/IPK pathway, and focusing predominantly on the unique aspects of fungi, we could be missing vital opportunities to expand our much needed antifungal armament to combat the predicted rise of IFIs in the future.

## Figures and Tables

**Table 1:** Summary of IP profiles of various strains [80,92,93]. In WT, + indicates that the species is present; in the mutants, multiple + indicates the level of accumulation of a species; − indicates the absence of a species. Note that IP_6_ is the most abundant IP species in WT (see Figure 2).

Strains	IP Species						
	IP_3_	IP_4_	IP_5_	PP-IP_4_	IP_6_	PP-IP_5_	PP_2_-IP_4_
WT H99	−	+	+	−	+	+	+
*arg1*Δ	+++	−	−	−	−	−	−
*ipk1*Δ	+	+	+++	+++	−	−	−
*kcs1*Δ	+	+	+	−	+	−	−
*asp1*Δ	+	+	+	−	+	+	−
*ipk1*Δ *kcs1*Δ	+	+	+++	−	−	−	−

**Table 2:** Phenotypes of cryptococcal IPK mutants (summarized from [75,80,92,93]). * refers to the unpublished data. N/A, the data is not available.

Mutant	*plc1*Δ	*arg1*Δ	*ipk1*Δ	*kcs1*Δ	*ipk1*Δ *kcs1*Δ	*asp1*Δ
Virulence in mice	Avirulent	N/A	Hypovirulent	Avirulent	Avirulent	Fully virulent
Virulence in invertebrate models	Hypovirulent in *C. elegans* (25 °C) and *G. mellonella* (30 °C)	Hypovirulent in *G. mellonella* (30 °C)	N/A	Hypovirulent in *G. mellonella* (30 °C)	N/A	N/A
Cell wall integrity	Compromised	Compromised	Compromised	Compromised	Compromised	Normal
Capsule production	Normal/reduced epending on growth conditions	Reduced	Normal *	Increased	Normal *	Normal
Urease production	Reduced	Reduced	Reduced	Reduced	Reduced	Normal
Mutant-specific features	Abnormally layered cell wall; large vacuoles; no septal dissolution	Abnormally thick cell wall; enlarged vacuoles; cell separation defect; accelerated endocytosis	Mucoid	Enlarged cell size; mucoid colony appearance	Mucoid	None
Carbon source utilization	N/A	N/A	Partially compromised	Defective	Defective	N/A
37 °C growth	Reduced	Reduced	Slightly reduced	Slightly reduced	Slightly reduced	Normal
Mating	Defective	Defective	Normal *	Defective	N/A	Normal
Melanization	Reduced	Reduced	Normal *	Reduced	Normal *	Normal
Laccase activity	Reduced *	Reduced *	Reduced	Reduced	Reduced	Normal *

**Table 3:** Examples of natural polyphenolic compounds that inhibit IPKs and also have antifungal activity. MIC: minimum inhibitory concentration; ED_50_: median effective dose.

Compound	Target Enzymes	Fungal Species/Inhibitory Concentration	Reference
Gossypol	Mammalian IP3K/IPMK	*Pythium irregulare* ED_50_ = 4.0 μg/mL *Rhizoctonia solani* ED_50_ = 34.6 μg/mL	[154]
Chlorogenic acid	Mammalian IPMK	*Candida albicans* MIC = 80 μg/mL *Trichosporon beigelii* MIC = 40 μg/mL *Malassezia furfur* MIC = 40 μg/mL	[155]
Quercetin	Mammalian IP3K	Synergistic effect with fluconazole (16 μg/mL) in fluconazole-resistant *Candida tropicalis* MIC_50_ ≤ 0.5 μg/mL	[156]
Ellagic acid	Mammalian IP3K/IPMK	*Trichophyton rubrum* MIC = 18.75 μg/mL *Trichophyton mentagrophytes* MIC = 32.29 μg/mL *Microsporum canis* MIC = 58.33 μg/mL *Candida albicans* MIC = 25 μg/mL *Candida tropicalis* MIC = 75 μg/mL	[157]
Hypericin	Mammalian IP3K	Natural photosensitizer, 3 log_10_ fungicidal effect at fluence of 37 J/cm^2^ *Candida albicans* 0.625 µM *Candida parapsilosis* 1.25 µM *Candida krusei* 20 μM *Trichophyton rubrum* 10–20 µM *Trichophyton mentagrophytes* 20–50 µM	[158] [159]
